# Mapping the Burden of Hypertension in Türkiye (2008–2022): A National Cross‐Sectional Study of Nearly 100000 Adults

**DOI:** 10.1155/ijhy/6660660

**Published:** 2026-06-18

**Authors:** Ridvan Sakir, Duha Yahya, Mehmet Kocak

**Affiliations:** ^1^ Department of Pathology, Kartal Dr. Lutfi Kirdar City Hospital, University of Health Sciences, Istanbul, Türkiye, uhs.edu.pk; ^2^ Pediatric Emergency Clinic, Basaksehir Cam ve Sakura City Hospital, Istanbul, Türkiye; ^3^ Health Sciences and Technologies Research Institute (SABITA), Istanbul Medipol University, Istanbul, Türkiye, medipol.edu.tr; ^4^ Department of Biostatistics and Medical Informatics, International School of Medicine, Istanbul Medipol University, Istanbul, Türkiye, medipol.edu.tr

**Keywords:** cross-sectional study, family history, hypertension, lifestyle, prevalence, public health, risk factors, Türkiye

## Abstract

**Background:**

Hypertension (HTN) remains one of the most common and modifiable risk factors for cardiovascular diseases worldwide. While several studies have explored its risk factors, updated national data covering long‐term trends in Türkiye has been limited.

**Methods:**

We conducted a cross‐sectional study using data from seven national health surveys carried out in Türkiye between 2008 and 2022, including a total of 97562 participants aged 19 and older. We calculated HTN prevalence across years and performed univariable and multivariable logistic regression analyses to examine associations with demographic, socioeconomic, and lifestyle factors.

**Results:**

Overall, 18.9% of participants reported to have HTN. Prevalence increased modestly from 2008 to 2016 and then stabilized by 2022 (18.4%). HTN was significantly associated with older age, female gender, higher BMI, larger waist circumference, lower education level, unemployment, physical inactivity, and having a spouse or parent with HTN. Notably, individuals with graduate‐level education had a higher prevalence of HTN than those without a high school education, and regular smokers showed a slightly lower prevalence compared to nonsmokers. There was also a significant regional difference in HTN diagnoses.

**Conclusion:**

Our findings offer an updated picture of HTN in Türkiye and highlight the influence of age, gender, lifestyle, geographical region, and family context on its prevalence. These results can guide future public health strategies of risk reduction at the individual, household, and regional levels.

## 1. Introduction

Hypertension (HTN) is a medical condition characterized by a persistent increase in systemic arterial pressure beyond a defined threshold. According to the European Society of Cardiology (ESC)/European Society of Hypertension (ESH), HTN is defined as blood pressure exceeding 140/90 mm Hg [1], whereas the American College of Cardiology (ACC) and American Heart Association (AHA)’s 2017 guidelines use a lower cutoff of 130/80 mm Hg [2]. HTN is the most frequently encountered condition in primary care settings and a mounting public health concern globally [3]. It is the leading modifiable risk factor contributing to cardiovascular disease burden and mortality on a global scale [4].

HTN significantly increases the risk of heart attacks, strokes, kidney failure, and mortality [5, 6]. It is the primary cause of death globally, responsible for at least 45% of fatalities from heart attacks and 51% from strokes [7]. HTN is responsible for more than 10 million deaths every year globally [8]. Despite this, it is estimated that 46% of adults with HTN are not aware that they have the condition [9], and only about 21% of adults have HTN effectively managed [9]. Approximately 1.28 billion (33%) adults between the ages of 30 and 79 worldwide are estimated to have HTN, with the majority (around two‐thirds) residing in low‐ and middle‐income countries [9]. Along the same lines, according to the 2023 WHO Global Report on HTN, an age‐standardized estimate indicates that around 33% of adults aged 30 to 79 years worldwide are affected by HTN [8]. In high‐income countries, around 30% of adults currently have HTN, and this figure is anticipated to rise to 60% over the next few decades [10]. Globally, HTN affects about 40.8% of the population, with regional variations such as 29.5% in Arab countries, a wide range of 9.3%–70.8% in Africa, 30.5% in Germany, and around the same rate among men in the United States, while the prevalence among American women is slightly lower, around 28.5% [11–14]. In other regions, HTN prevalence rates are reported as 41.8% in Iran, 29.8% in India, 29.6% in China, and 30.8% among South Asian immigrants residing in the United Arab Emirates (UAE), indicating significant geographical variance [15]. HTN is a significantly higher disease burden in low‐income countries, leading to cardiovascular disease mortality of up to 80% [16].

Decreased physical activity combined with increased calorie consumption from unhealthy diets has contributed to a greater prevalence of clinical risk factors, including HTN, overweight, obesity, dyslipidemia, and diabetes [17]. A meta‐analysis by Han et al., which included 47 cohort studies and 491666 participants, showed that effective management of pre‐HTN led to a reduction of over 10% in cardiovascular disease, coronary heart disease, heart attacks, and strokes [18].

In Türkiye, HTN remains a serious public health issue. As of 2019, approximately 33% of the population, about 13.8 million adults, are hypertensive, including 31% of men and 34% of women [8], quite in line with the global picture. The regional variation of HTN prevalence reported by Mills et al. for the global population [6, 11, 19] has been also reported among the subregions of Türkiye as well [20]. Similarly, a global increase in the proportion of the aging population compared to the pediatric and working‐adult population is a reality for Türkiye as well [21], which results in a steady increase of HTN prevalence. As a result of these, HTN is responsible for 432000 deaths per year as of 2019 in Türkiye [8]. Several modifiable risk factors contribute to this burden: 31% of adults over 18 are physically inactive, 32% are obese, 31% of individuals aged 15 years and above use tobacco, and average daily salt intake among adults over 25 is 5 g [8]. In Türkiye, alcohol consumption among adults 15 years and older is 2 L per capita annually [8]. While some population‐based studies have found a positive link between chronic smoking and HTN [22], others have reported no significant association [23, 24]. Better understanding the risk factors of HTN is essential to generate more timely prevention and treatment strategies not only for HTN but also for its sequelae.

In this study, we use data from a series of seven independent national health surveys conducted in 2008, 2010, 2012, 2014, 2016, 2019, and 2022 to examine the prevalence of HTN among adults in Türkiye. Thus, this study includes a series of cross‐sectional analyses where we explored how different sociodemographics and behavioral factors—like age, gender, education, exercise, smoking, geographical region, and body measurements—are associated with HTN. Our goal is to provide an updated picture of HTN in Türkiye over a 14‐year period and highlight areas that might aid public health policy development.

## 2. Materials and Methods

The Turkish Health Survey (THS) was first conducted in 2008 and was implemented in the years of 2010, 2012, 2014, 2016, 2019, and 2022 by the Turkish Statistical Institute (TSI). It adopted modules from the Eurostat European Health Interview Survey (EHIS) and was conducted as a part of EHIS waves in Europe along with Türkiye.

The data collection technique has been detailed in earlier literature [25–27]. A stratified two‐stage cluster sampling method was used for the survey. The stratification was based on distinguishing between rural and urban areas. In the first stage, clusters (blocks) were randomly selected with probabilities proportional to their size. In the second stage, a random and systematic selection of household addresses was carried out within each selected cluster. The sampling covered all regions across Türkiye and included all residents, excluding those in institutional environments—such as military bases, dormitories, long‐term care hospitals, and nursing homes—and in very small settlements that could not yield enough sample households (e.g., small villages). The sample size ranged from 7910 households in 2008 to 11179 in 2022. Data collection was done through face‐to‐face interviews, starting in April 2008. Follow‐up surveys were conducted during May–June in 2010 and 2012, August–October in 2014 and 2016, and September–December in 2019 and 2022. The survey had sample sizes sufficiently representative at the 12 Nomenclature of Territorial Units for Statistics (NUTS) level 1 regions.

The overall aims of the surveys were to regularly gather data on sociodemographics, behavior, access and utilization of healthcare, and health indicators in Türkiye to track trends in the population’s health and the factors influencing it. In line with Eurostat guidelines, the survey had maintained a consistent focus on individuals aged 0–14 and 15 years and older, even though some questions have evolved over time. For participants aged 15 years and above, the survey is structured around three main sections: health status, healthcare services, and health‐related behaviors. The health status section includes information on chronic illnesses, diseases, injuries, physical and sensory disabilities, daily personal care challenges, pain, and mental health conditions. The healthcare services section addresses the use of inpatient and outpatient care, medication use, and preventive healthcare. The section on health determinants gathers data on body measurements (such as weight and height), levels of physical activity, eating habits, and consumption of alcohol and tobacco.

Participants reported their chronic conditions by selecting from the options: “Yes,” “No,” “Do not know,” or “Refuse to answer.” Participants also had the choice to skip individual questions. The specific question for HTN was “During the past 12 months, have you had high blood pressure (hypertension)?” and the answer of “Yes” or “No” indicated the status of a participant having or not having HTN, and this indicator variable was defined as the primary outcome variable as a participant‐reported outcome, and those who did not answer the question or answered as, “Do not know,” or “Refuse to answer” were removed.

Regional classifications were based on the NUTS1 regions, which segments the country into large geographics and socioeconomic areas. Educational attainment was categorized following the ISCED 2011 framework and Eurostat recommendations and classified as “< high school,” “high school,” “undergraduate,” and “graduate” education. Body mass index (BMI) was categorized into four groups: underweight (BMI < 18.5), normal weight (BMI 18.5–24.9), overweight (BMI 25.0–29.9), and obese (BMI ≥ 30.0). Physical activity measures were asked as the number of days in a typical week. For example, for walking, the exact phrasing of the question was “In a typical week, on how many days do you walk for at least 10 min continuously in order to get to and from places?” We defined the following question to represent moderate physical activity: “In a typical week, on how many days do you carry out sports, fitness or recreational (leisure) activities for at least 10 min continuously?”, and the following question to represent vigorous physical activity: “In a typical week, how many days do you carry out activities specially designed to strengthen your muscles such as doing resistance training or strength exercises?” All lifestyle indicators and measures are self‐reported by participants as expected.

As the national survey was conducted on family clusters and provided the relatedness indicators of participants, we also wanted to carry out a subset analysis utilizing the information of disease conditions within the family, like a spouse having the same conditions, or mother and father of the primary respondent having the same conditions, and we constructed separate models to investigate the association of these familial factors as well.

In variable selections, we have conducted univariable analyses through chi‐square tests for categorical predictors and Wilcoxon–Mann–Whitney tests for continuous predictors. We then utilized multivariable logistic regression models to examine the association of promising variables, including time, geographical regions, sociodemographics, anthropometric and behavioral factors, and other health indicators, with the likelihood of HTN. We included the NUTS‐1 region as a categorical factor in our models due to the earlier reports on regional variability in HTN prevalence [6, 11, 19]. As these analyses were carried out with a hypothesis‐generating context, a *p*‐value of less than 0.05 was considered statistically significant throughout the study. Statistical analysis and visualizations were conducted in SAS ® Version 9.4 (Cary, North Carolina, USA).

## 3. Results

Our final analysis sample included 97562 participants aged over 18 who had available HTN status data from seven national health surveys conducted in Türkiye between 2008 and 2022. Out of all participants, 22789 (18.9%) were found to have HTN. The prevalence of HTN increased slightly between 2012 and 2016, going from 17.4% to 20.8%. In 2022, it settled at 18.4%, which is just a bit higher than the 18.2% reported back in 2008 (Table 1).

**TABLE 1:** Hypertension prevalence over time.

	**Participants with hypertension**	
	**Number**	**Prevalence (with 95% confidence interval)**

All participants	22789	18.9 (18.4, 19.4)
2008	2379	18.2 (16.6, 19.8)
2010	2289	17.8 (16.2, 19.4)
2012	4366	17.4 (16.3, 18.5)
2014	3523	20.4 (19.1, 21.7)
2016	3251	20.8 (19.4, 22.2)
2019	3159	20.1 (18.7, 21.5)
2022	3822	18.4 (17.2, 19.6)

There was a clear age‐related pattern in HTN prevalence, with rates increasing steadily as age went up. Among participants aged 20–29, only 1.5% had HTN. This rose to 4.4% in those aged 30–39, 12.5% in the 40–49 group, and 28% in participants aged 50–59. Prevalence continued to rise with age—reaching 44.6% in the 60–69 age group, 54.6% in those aged 70–79, and peaking at 57.2% among participants aged 80 and above. The association between age and HTN was statistically significant (*p* < 0.0001).

Gender differences were also highly significant among all participants; 13.3% of males had HTN, compared to 23.3% of females.

Individuals without a high school education had the highest prevalence (23.5%), followed by high school graduates (9.2%) and undergraduates (7.8%). Interestingly, participants with graduate‐level education showed a higher prevalence of HTN (25.8%).

Regular smokers had a lower prevalence of HTN (16.3%) compared to nonregular smokers (18.3%). In this study, regular smoking was defined as using tobacco products every day or almost every day for at least 1 year. This seeming association may be perhaps due to confounding with the younger ages of smokers.

HTN prevalence varied significantly by marital status. Divorced individuals had the highest rate (49.5%), followed by widowed (37.8%) and married individuals (18.7%). In contrast, single individuals showed the lowest prevalence (2.6%), again perhaps due to the confounding with expected younger ages of singles.

HTN prevalence also differed by employment status, with unemployed individuals experiencing a much higher rate (26%) than their employed counterparts (8.6%).

There was a clearly significant association of geographical region with HTN prevalence, with the highest rates observed in Western Anatolia (26.7%) and the Aegean Region (25%), followed by Eastern Marmara (22.8%), Middle Anatolia (21%), and Middle Eastern Anatolia (20%). Lower prevalence was found in Istanbul (19.7%), the Mediterranean Region (19.5%), and Southeastern Anatolia (18.3%), while the lowest rates occurred in Northeastern Anatolia (17.6%), Western Marmara (17.5%), the Western Black Sea Region (15%), and the Eastern Black Sea Region (14.3%).

In addition to the above significant factors, participants aged ≥ 19 years, older age, higher body weight, and more years of smoking were associated with increased odds of HTN as anticipated. Conversely, taller height and a higher level of engagement in physical activity—including moderate, vigorous, or even walking for at least 10 min daily—were linked to significantly lower odds of HTN, univariably (Table 2).

**TABLE 2:** Association of continuous variables with hypertension diagnosis (all markers have significantly different distributions by hypertension status, *p*‐values < 0.0001).

		N	Min	Q1	Med	Q3	Max	Mean	SD
Age	Participants without hypertension	97562	20.0	30.0	40.0	51.0	105.0	41.9	14.6
	Participants with hypertension	22789	20.0	51.0	61.0	70.0	120.0	60.5	13.4

Height (cm)	Participants without hypertension	93840	100.0	160.0	167.0	174.0	210.0	167.5	8.9
	Participants with hypertension	21343	52.0	158.0	164.0	170.0	198.0	163.6	8.6

Weight (kg)	Participants without hypertension	95205	20.0	63.0	72.0	82.0	190.0	73.1	14.1
	Participants with hypertension	21952	23.0	68.0	78.0	87.0	170.0	78.0	14.9

BMI	Participants without hypertension	93391	12.3	22.9	25.6	28.7	64.9	26.1	4.6
	Participants with hypertension	21225	13.6	25.5	28.7	32.4	66.4	29.2	5.4

Years of tobacco smoking	Participants without hypertension	97562	0.0	0.0	0.0	0.0	80.0	2.4	7.7
	Participants with hypertension	22789	0.0	0.0	0.0	0.0	72.0	2.8	9.4

No. of days in a typical week with moderate exercise	Participants without hypertension	96068	0.0	0.0	0.0	0.0	7.00	0.50	1.64
	Participants with hypertension	22560	0.0	0.0	0.0	0.0	7.00	0.36	1.40

No. of days in a typical week with vigorous exercise	Participants without hypertension	96595	0.0	0.0	0.0	0.0	7.00	0.23	1.12
	Participants with hypertension	22644	0.0	0.0	0.0	0.0	7.00	0.11	0.78

No. of days in a typical week with walking 10 m/day	Participants without hypertension	79232	0.0	0.0	5.00	7.00	7.00	3.89	3.01
	Participants with hypertension	18733	0.0	0.0	3.00	7.00	7.00	3.15	3.03

Abbreviations: Max: maximum; Med: median; Min: minimum; N: number; Q1: Quarter 1; Q3: Quarter 3; SD: standard deviation.

On these univariably significant predictors, we constructed a multivariable logistic regression model with stepwise variable selection, resulting in an AUC of 0.835 (Table 3). When compared to the oldest age group (80+ years), all younger age groups showed statistically significant differences (*p* < 0.0001). Temporal trends indicated that most survey years (2010, 2012, 2016, 2019, and 2022) had lower odds of HTN compared to 2008, with the exception of 2014, which showed slightly elevated odds (OR: 1.03, *p* < 0.0001).

**TABLE 3:** Multivariable results of the likelihood of hypertension (participants with > 19 years of age).

Effect	Odds ratio (95% CI)	*p*‐value
Age in [20,29) (ref = age in [80,+))	0.01 (0.01, 0.02)	< 0.0001
Age in [30,39) (ref = age in [80,+))	0.04 (0.04, 0.05)	< 0.0001
Age in [40,49) (ref = age in [80,+))	0.13 (0.12, 0.14)	< 0.0001
Age in [50,59) (ref = age in [80,+))	0.34 (0.31, 0.37)	< 0.0001
Age in [60,69) (ref = age in [80,+))	0.66 (0.61, 0.72)	< 0.0001
Age in [70,79) (ref = age in [80,+))	0.95 (0.88, 1.04)	< 0.0001
Year 2010 (ref = 2008)	0.85 (0.79, 0.92)	0.0006
Year 2012 (ref = 2008)	0.84 (0.79, 0.90)	< 0.0001
Year 2014 (ref = 2008)	1.03 (0.96, 1.11)	< 0.0001
Year 2016 (ref = 2008)	0.97 (0.90, 1.04)	0.039
Year 2019 (ref = 2008)	0.95 (0.88, 1.02)	0.36
Year 2022 (ref = 2008)	0.87 (0.81, 0.93)	0.0006
Female (ref = male)	2.02 (1.95, 2.10)	< 0.0001
High school (ref=< high school)	0.79 (0.74, 0.83)	< 0.0001
Undergraduate < high school	0.71 (0.67, 0.76)	< 0.0001
Graduate < high school	1.02 (0.92, 1.12)	< 0.0001
Employment status: yes (ref = no)	0.72 (0.69, 0.75)	< 0.0001
Northeastern Anatolia (ref = Istanbul)	0.84 (0.74, 0.94)	0.005
Middle Eastern Anatolia (ref = Istanbul)	0.98 (0.90, 1.06)	0.9
Southeastern Anatolia (ref = Istanbul)	1.00 (0.91, 1.11)	0.43
Western Marmara (ref = Istanbul)	0.92 (0.86, 0.99)	0.047
Aegean Region (ref = Istanbul)	1.12 (1.04, 1.21)	< 0.0001
Eastern Marmara (ref = Istanbul)	1.07 (0.98, 1.16)	0.005
Western Anatolia (ref = Istanbul)	1.21 (1.10, 1.34)	< 0.0001
Mediterranean (ref = Istanbul)	0.96 (0.89, 1.03)	0.61
Middle Anatolia (ref = Istanbul)	0.94 (0.88, 1.00)	0.14
Western Black Sea (ref = Istanbul)	0.80 (0.74, 0.87)	< 0.0001
Eastern Black Sea (ref = Istanbul)	0.89 (0.83, 0.94)	< 0.0001

Abbreviations: AUC: area under the curve; CI: confidence Interval; N: number.

Controlling for other significant predictors, women had more than double the odds of HTN compared to men (OR: 2.02, *p* < 0.0001). Educational attainment showed a complex pattern: both high school (OR: 0.79) and undergraduate education (OR: 0.71) were protective compared to not completing high school, while graduate education showed marginally higher odds (OR: 1.02, *p* < 0.0001). Employment status was also significant, with employed individuals showing 28% lower odds than their unemployed counterparts (OR: 0.72, *p* < 0.0001).

Geographic variations were significantly different multivariable as well; when comparing with the Istanbul Region, several regions showed significantly lower odds, including the Western Black Sea (OR: 0.80), Northeastern Anatolia (OR: 0.84, *p* = 0.005), and Eastern Black Sea (OR: 0.89). Other regions like Western Anatolia (OR: 1.21) and the Aegean (OR: 1.12) demonstrated higher odds. The other regions hovered near the reference value for Istanbul, with odds ratios ranging from 0.92 to 1.07.

When we restricted the multivariable analysis to adults aged 39+ (Table 4), age, gender, education level, and employment status remained statistically significant predictors of HTN. The regional patterns showed reduced distinctiveness among older adults. Several previously significant regional differences became nonsignificant: Middle Eastern (OR 1.02, *p* = 0.56), Southeastern (OR 1.05, *p* = 0.29), and Mediterranean (OR 0.98, *p* = 0.4) regions now showed comparable prevalence to Istanbul. Western Marmara (OR 0.95, *p* = 0.066) and Middle Anatolia (OR 0.97, *p* = 0.16) showed marginally lower, though not statistically significant, odds compared to Istanbul.

**TABLE 4:** Multivariable results of the likelihood of hypertension (participants with > 39 years of age).

Effect	Odds ratio (95% CI)	*p*‐value
Age in [20,29) (ref = age in [80,+))	0.13 (0.12, 0.14)	< 0.0001
Age in [30,39) (ref = age in [80,+))	0.34 (0.31, 0.36)	< 0.0001
Age in [40,49) (ref = age in [80,+))	0.66 (0.61, 0.72)	< 0.0001
Age in [50,59) (ref = age in [80,+))	0.96 (0.88, 1.04)	< 0.0001
Age in [60,69) (ref = age in [80,+))	0.87 (0.81, 0.95)	0.0003
Age in [70,79) (ref = age in [80,+))	0.90 (0.83, 0.97)	0.0006
Year 2010 (ref = 2008)	1.06 (0.99, 1.15)	< 0.0001
Year 2012 (ref = 2008)	1.01 (0.93, 1.09)	0.022
Year 2014 (ref = 2008)	0.98 (0.91, 1.06)	0.29
Year 2016 (ref = 2008)	0.90 (0.84, 0.97)	0.003
Year 2019 (ref = 2008)	2.08 (2.00, 2.17)	< 0.0001
Year 2022 (ref = 2008)	0.83 (0.78, 0.88)	0.0007
Female (ref = male)	0.79 (0.74, 0.85)	< 0.0001
High school (ref=< high school)	1.04 (0.93, 1.16)	0.002
Undergraduate < high school	0.72 (0.68, 0.75)	< 0.0001
Graduate < high school	0.85 (0.75, 0.97)	0.005
Employment status: yes (ref = no)	1.02 (0.93, 1.12)	0.56
Northeastern Anatolia (ref = Istanbul)	1.05 (0.95, 1.16)	0.29
Middle Eastern Anatolia (ref = Istanbul)	0.95 (0.88, 1.02)	0.066
Southeastern Anatolia (ref = Istanbul)	1.15 (1.06, 1.25)	< 0.0001
Western Marmara (ref = Istanbul)	1.10 (1.01, 1.20)	0.007
Aegean Region (ref = Istanbul)	1.29 (1.16, 1.43)	< 0.0001
Eastern Marmara (ref = Istanbul)	0.98 (0.91, 1.05)	0.4
Western Anatolia (ref = Istanbul)	0.97 (0.91, 1.03)	0.16
Mediterranean (ref = Istanbul)	0.82 (0.75, 0.90)	< 0.0001
Middle Anatolia (ref = Istanbul)	0.91 (0.86, 0.98)	< 0.0001
Western Black Sea (ref = Istanbul)	0.13 (0.12, 0.14)	< 0.0001
Eastern Black Sea (ref = Istanbul)	0.34 (0.31, 0.36)	< 0.0001

Abbreviations: AUC: area under the curve; CI: confidence Interval; N: number.

Controlling for other known factors of HTN, the presence of HTN in one’s spouse showed a significant association with the reference person’s HTN status across all age groups. When the spouse does not have HTN, it reduces one’s odds of having HTN by around 34% (OR (95% CI): 0.66 (0.62, 0.71), *p* < 0.0001), after controlling for other key predictors of HTN. In addition, a parental history of HTN significantly increased HTN risk in the reference person. Individuals without hypertensive parents had substantially lower odds of HTN compared to those with hypertensive parents (OR (95% CI): 0.34 (0.23, 0.49), *p* < 0.0001).

## 4. Discussion

Based on a series of seven national surveys conducted between 2008 and 2022, this study aimed to provide an overview of the trajectory of HTN and its associated risk factors in Türkiye.

Our multivariable models showed that the prevalence of HTN in Türkiye showed a slight increase over the past decade, followed by a period of stabilization in recent years. By 2022, the prevalence had reached 18.4%, which was nearly identical to the 2008 level (Table 1). This figure is lower than reported rates in many other countries, including most African nations, the United States, Germany, Iran, China, and India [12–15].

**FIGURE 1:**
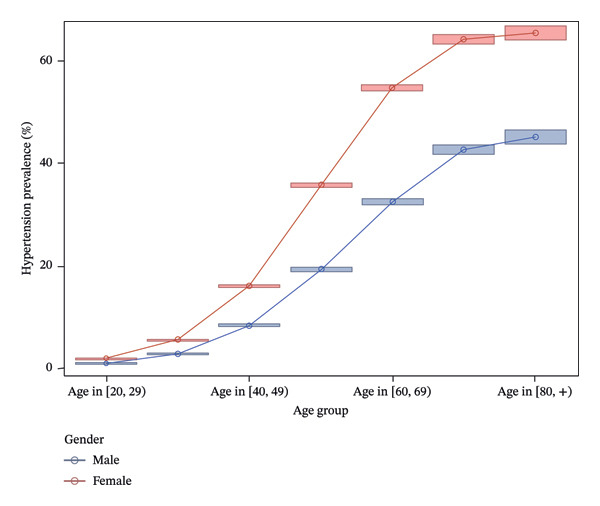
Age‐related prevalence of hypertension by gender across years. The height of bars shows the standard error of the prediction of hypertension.

A strong age‐related trend was observed, with HTN prevalence increasing steadily with age (Figure 1). Only 1.5% of participants aged 20–29 had HTN, compared to 57.2% among those aged 80 and above. This pattern aligns with findings from previous studies [13, 28–30].

We found significantly higher female HTN prevalence compared to males, with 2 times increased risk. Although increased female prevalence has been reported in some studies [28], the magnitude of this difference in our study was much more pronounced. In contrast, several global studies have reported higher HTN prevalence in men, particularly in younger populations [31, 32]. Global evidence suggests that sex differences in HTN are heterogeneous and often age‐dependent, with men showing higher prevalence at younger ages and women surpassing men at older ages [33, 34].

One possible explanation for our finding is the higher level of HTN awareness, diagnosis, and treatment among women. Previous studies have shown that women are more likely to be aware of their hypertensive status and to seek healthcare, which may lead to higher reported prevalence due to increased detection rather than true underlying risk [35].

A study conducted in Türkiye reported a 1.7‐fold increase in HTN prevalence among females, particularly in urban settings [36], which may partially explain our findings. However, the stronger association observed in our study suggests that local population characteristics and healthcare access patterns may have contributed to this difference, and therefore, the observed female predominance should be interpreted within the specific sociodemographic context of Türkiye.

Education level showed an inverse relationship with HTN prevalence in our study, with one exception at the graduate education level. Previous studies have consistently reported that HTN prevalence tends to decrease as education level increases [36, 37]. Similarly, we observed a decreasing trend in HTN prevalence up to the undergraduate level. However, participants with graduate‐level education had a higher prevalence of HTN than those without a high school education (OR: 1.02, *p* < 0.0001), which was an unexpected finding.

One possible explanation for this observation is age distribution, as individuals with graduate‐level education may be older on average. In addition, occupational stress, sedentary work patterns, and lifestyle factors commonly associated with higher educational attainment may contribute to increased HTN risk [38]. Furthermore, individuals with higher education levels may have greater access to healthcare services and improved HTN detection and management, leading to higher reported diagnosis rates [39].

To address potential confounding, we evaluated this association in multivariable models adjusting for age and other relevant covariates. After adjustment, the association between graduate‐level education and HTN was attenuated and became nonsignificant in the overall population. However, in age‐stratified analyses, graduate‐level education was associated with a lower risk of HTN among older individuals. These findings suggest that the initially observed higher prevalence among graduate‐level individuals may be largely explained by confounding factors, particularly age, and that the effect of education on HTN may vary across age groups. Therefore, this finding should be interpreted with caution.

We observed that both reference persons and their spouses had similar rates of HTN, whereas first‐degree relatives had considerably lower prevalence. Although direct comparisons by family role are not commonly explored in the literature, this difference is likely attributable to age, as first‐degree relatives in household surveys often include younger individuals such as children or adolescents. The similarity between reference persons and spouses may reflect shared environmental or lifestyle factors within households.

An unexpected finding in our study was that regular smokers had a lower prevalence of HTN (16.3%) compared to nonregular smokers (18.3%). While some studies have reported an inverse relationship between regular smoking and HTN, others have shown mixed results—often finding no significant association in current smokers but an increased risk among former smokers [23]. These discrepancies are likely influenced by confounding factors such as age, body weight, and general health status, which may vary between smoking categories and affect HTN risk independently.

Marital status was another significant factor in our study. We found that divorced individuals had the highest prevalence of HTN, followed by widowed and then married individuals. The lowest prevalence was observed among single participants, which is consistent with previous studies [15]. The elevated rates among divorced and widowed individuals may be partly explained by older age, as well as the psychological stress and emotional burden associated with these life circumstances.

Employment status was also associated with HTN prevalence in our study. Unemployed individuals had a significantly higher prevalence (26%) compared to those who were employed (8.6%), which aligns with findings from previous studies [36, 40]. This difference suggests that economic stability, social structure, and routine associated with employment may have protective effects against HTN.

Regionally, HTN prevalence varied significantly across Türkiye. The highest rates were observed in the Western Anatolia Region (26.7%) and the Aegean Region (25%), while the lowest rates were found in the Western and Eastern Black Sea Regions (14.3% and 15%, respectively). These findings contrast with a previous study from Türkiye, which reported the highest prevalence in the Black Sea Region and the lowest in the Mediterranean [23]. Such differences may be explained by variations in sample size, population characteristics, or methodological approaches between studies.

In the univariable logistic regression analysis for adults aged 19 and older, several factors were associated with increased odds of HTN, including older age, higher body weight, greater waist circumference, and a greater number of years spent smoking. These findings are consistent with previous studies [28, 41]. Conversely, taller individuals had lower odds of HTN, which aligns with findings from a study conducted in Indonesia [42]. This may be explained by the fact that, for a given weight, taller individuals have a lower BMI. Those engaging in physical activity—whether moderate, vigorous, or walking at least 10 min a day—had significantly lower odds of having HTN, which aligned with previous studies [25, 26]. Additionally, males had approximately half the odds of having HTN compared to females (OR = 0.52).

Multivariable logistic regression analysis revealed that increasing age and being female were significantly associated with higher odds of HTN. In contrast, being employed and having an undergraduate education (compared to having less than a high school education) were linked to lower odds. These findings reinforce the role of demographic, socioeconomic, and lifestyle factors in shaping HTN risk. Additionally, significant differences were observed across regions and survey years, suggesting that both geographic and temporal variations may influence the burden of HTN in Türkiye.

One of the key findings of our study was that having a spouse with HTN significantly increased the risk of HTN in the reference individual. This association was also reported in a previous study involving populations from the United States, England, China, and India, which supports our results [43]. This relationship may be explained by shared environmental exposures and behavioral concordance between spouses, including similar dietary habits, physical activity patterns, and lifestyle factors, which are known to influence HTN risk [43].

Another important finding from our study was that having a mother or father with HTN was associated with an increased risk of HTN in the reference individual, highlighting the importance of family history as a risk factor. This finding is consistent with previous studies examining the role of family history in HTN [15, 37]. The observed association may reflect both genetic predisposition and shared environmental influences within families.

To our knowledge, this is the first study to examine both spousal and parental HTN associations in a Turkish population, providing novel insights into the combined effects of familial and household‐level risk factors in this context.

One of the major strengths of our study is its large, nationally representative sample, including nearly 100000 individuals from across Türkiye. This provides a strong foundation for generalizing our findings to the broader adult population. Additionally, although our design is cross‐sectional, the inclusion of seven national surveys spanning 14 years allowed us to observe changes in HTN prevalence and related risk factors over time. The study also included a wide range of demographic, lifestyle, and anthropometric variables, which gave us a chance to look at HTN from multiple angles. The use of a multivariable regression model helped strengthen the reliability of our associations by adjusting for potential confounders.

Another important strength is the originality of our findings. To our knowledge, this is the first study in Türkiye to evaluate the impact of having a hypertensive mother or father separately and also to look at how a spouse’s HTN status may influence individual risk.

That said, there are also a few limitations worth noting. Since the study is cross‐sectional, we can only identify associations—not causation. Much of the data, including HTN status, physical activity, smoking, and family history, was self‐reported, which could introduce recall bias. We were also not able to include other relevant factors like dietary habits, stress levels, or genetic background, which might influence HTN risk. While we observed regional differences, these may be partially shaped by sampling variation or differences in regional population structure that we could not fully adjust for, such as dietary differences among regions, salt intake, and physical activity limitations, as these measures are self‐reported and expected to have depth limitations within the context of a national survey. Another limitation in statistical modeling is that the survey weights were not accessible, thus not utilized in our multivariable logistic regression models.

## 5. Conclusion

In conclusion, this study provides a comprehensive look at HTN trends and risk factors in Türkiye over the past 14 years. Our findings confirm the continued impact of age, gender, education, lifestyle, and family history on HTN risk. We also identified unique associations related to marital and family roles that have not been widely studied before in the Turkish population. These insights can help guide future public health efforts by focusing on individual, household, and regional‐level risk factors. Continued monitoring through more in‐depth national surveys, especially in lifestyle variables and targeted public health policies and interventions, will be key to managing the burden of HTN in the country.

## Author Contributions

Ridvan Sakir generated the research idea, carried out the literature search, and wrote the initial and final draft of the manuscript; Duha Yahya helped with the literature search, manuscript writing, and detailing the discussions; and Mehmet Kocak acquired the research data, carried out the data analyses and modeling, provided the materials and methods section of the manuscript, and approved the final manuscript.

## Funding

Partial financial support was received from the TUBITAK Directorate of Science Fellowships and Grant Programmes (BIDEB)‐2232 International Fellowship for Outstanding Researchers (award no. 118C306).

## Disclosure

The opinions raised in this article solely belong to its authors and do not represent the position of TUBITAK and TSI in any shape or form.

## Ethics Statement

Our research protocol was approved by Istanbul Medipol University Ethics Committee (application number: 10840098‐604.01.01‐E.53819). The Ethics Committee waived the need for informed consent, as there is no human subject involved in this research. Data are simply province‐level mortality data provided by the Turkish Statistical Institute per year.

## Consent

The authors have nothing to report.

## Conflicts of Interest

The authors declare no conflicts of interest.

## Data Availability

As the death records data utilized in this report were granted access only to the corresponding author, we do not have the permission to share these data components; however, we can share the environmental data upon request. Please contact Dr. Mehmet Kocak at mehmetkocak@medipol.edu.tr regarding such data requests.
